# Do Extracellular RNAs Provide Insight into Uveal Melanoma Biology?

**DOI:** 10.3390/cancers13235919

**Published:** 2021-11-25

**Authors:** Cristina Barbagallo, Chiara Bianca Maria Platania, Filippo Drago, Davide Barbagallo, Cinzia Di Pietro, Michele Purrello, Claudio Bucolo, Marco Ragusa

**Affiliations:** 1 Department of Biomedical and Biotechnological Sciences—Section of Biology and Genetics, University of Catania, 95123 Catania, Italy; cbarbagallo@unict.it (C.B.); dbarbaga@unict.it (D.B.); dipietro@unict.it (C.D.P.); purrello@unict.it (M.P.); mragusa@unict.it (M.R.); 2 Department of Clinical and Experimental Medicine, University of Catania, 95123 Catania, Italy; 3 Department of Biomedical and Biotechnological Sciences—Section of Pharmacology, University of Catania, 95123 Catania, Italy; chiara.platania@unict.it (C.B.M.P.); f.drago@unict.it (F.D.); 4 Center of Research in Ocular Pharmacology—CERFO, University of Catania, 95123 Catania, Italy

**Keywords:** UM, miRNA, mRNA, lncRNA, circulating RNA, RNA carrier, cancer, eye

## Abstract

**Simple Summary:**

The study of RNAs in the extracellular environment in physiological and pathological conditions has become a growing field of research with intriguing applications in diagnostics and prognostics. Such extracellular RNAs are passively or actively released by all cells into biological fluids to spread biological signals to other cells. The perturbation of such RNA-based cell-to-cell communications in cancer can be easily identified by molecular analysis of liquid biopsies, even if source cells secreting RNAs are often elusive. In uveal melanoma (UM), extracellular RNAs can be assayed in serum, plasma, and vitreous and aqueous humor. In this review, we explore the possibility that extracellular RNA alterations in UM could partially match with RNA dysregulations observed in tumor tissues and provide information to better understand UM biology.

**Abstract:**

Uveal melanoma (UM) is the most common primary intraocular malignant tumor in adults, showing a high mortality due to metastasis. Although it is considered a rare disease, a growing number of papers have reported altered levels of RNAs (i.e., coding and non-coding RNAs) in cancerous tissues and biological fluids from UM patients. The presence of circulating RNAs, whose dysregulation is associated with UM, paved the way to the possibility of exploiting it for diagnostic and prognostic purposes. However, the biological meaning and the origin of such RNAs in blood and ocular fluids of UM patients remain unexplored. In this review, we report the state of the art of circulating RNAs in UM and debate whether the amount and types of RNAs measured in bodily fluids mirror the RNA alterations from source cancer cells. Based on literature data, extracellular RNAs in UM patients do not represent, with rare exceptions, a snapshot of RNA dysregulations occurring in cancerous tissues, but rather the complex and heterogeneous outcome of a systemic dysfunction, including immune system activity, that modifies the mechanisms of RNA delivery from several cell types.

## 1. Introduction

The study of extracellular RNAs represents a current area of research with intriguing potential applications for diagnostic and prognostic procedures. Contrary to what people might think, the presence of RNA molecules circulating in biological fluids has been known for a long time. The first papers demonstrating that RNA is present in media collected from cultured mammalian cells date back to the 1970s [1,2]. However, such a phenomenon was not further studied and largely ended up on the scrap heap. The presence of RNAs in the extracellular environment has been re-evaluated almost 40 years after the original discovery, and, in only a few years, it has become a growing field of research for basic and applied biosciences, capturing the imagination of the scientific community. Based on the idea that RNAs transcribed within a cell (the ‘donor’ cell) are released into the extracellular environment and internalized into recipient cells [1], several reports have shown that RNAs can be exchanged among cells and play an unanticipated role in cell-to-cell communication [3,4,5,6]. It has been shown that such RNA signaling could be perturbed by pathological conditions, such as cancer [7,8], leading to an altered stochiometric concentration of RNA molecules circulating in bodily fluids, including plasma, serum, urine, saliva, semen, vitreous humor, ascites, and cerebrospinal fluid [9,10,11,12,13,14].

Circulating RNAs include several subtypes of molecules, such as messenger RNAs (mRNAs), long non-coding RNAs (lncRNAs), as well as various species of small non-coding RNAs (ncRNAs) including microRNAs (miRNAs), piwi-interacting RNAs (piRNAs), small interfering RNAs (siRNAs), tRNA-derived fragments, and Y RNAs [15,16,17,18].

Several studies proved that RNAs are thrown into the circulation in different ways. Part of the extracellular RNAs is the consequence of the passive leakage of apoptosis, necrosis, or inflammation mechanisms, while other parts are actively secreted within nano/micro-vesicles, lipoproteins, and ribonucleoprotein complexes [19]. When cell death occurs, cytoplasmatic fragments from dying cells are trapped inside the apoptotic bodies, which are swallowed by neighboring living cells via phosphatidylserine signaling. By this passive mechanism, several miRNAs are transported into the apoptotic bodies, and then potentially released into the circulation and captured by recipient cells [20]. There are multiple types of extracellular vesicles, differentiated by their size, biogenesis, releasing mechanism, and cargo, which are involved in active mechanisms of RNA secretion. The main type of vesicles includes microvesicles (100–1000 nm in size), deriving from blebbing of the plasma membrane, and exosomes (30–100 nm in size), originating from endosomal bodies [21]. Finally, non-vesicle-associated RNA carriers include ribonucleoprotein (RNP) complexes, such as Argonaute2, GW182, nucleophosmin 1, and high-density lipoproteins [22,23,24,25]. Interestingly, extracellular DNA can be detected in bodily fluids in higher concentrations than extracellular RNA [26,27]. Different DNA-releasing mechanisms have been proposed, such as apoptosis, necrosis, active release, and exocytosis, and recently some papers reported the association of extracellular DNA with uveal melanoma diagnosis and prognosis [28,29,30,31]. However, we will not discuss how circulating DNA reflects uveal melanoma biology because the functioning of active releasing mechanisms of DNA (e.g., vesicles and virtosomes) remains elusive and debatable even now [27,32].

Regardless of the nature of RNA carriers, RNA molecules are packaged, transported into the extracellular environment, protected from RNase degradation, and delivered to recipient cells, where they can influence cellular functions [3,16,23,33]. The RNA content changes according to the donor cell type [33], and noticeable qualitative and quantitative differences have been reported between cancer cells and their physiological counterparts [34,35,36]. This growing number of experimental observations, including eye cancer [12,37,38,39] and retinal degeneration [40,41,42,43,44], paved the way for extracellular RNA exploitation for diagnostic and prognostic purposes [45]. However, the real biological meaning of such RNA signaling among cells in physiological and pathological conditions remains slippery. Most importantly, the functional relationship between secreted RNAs and those retained in the cytoplasm remains unclear in tumoral phenotypes [4]. Regarding liquid biopsy applications in clinical management, an understanding whether the amount and types of RNAs measured in bodily fluids mirror the RNA alterations within cancer donor cells is needed. In other words, how much does the RNA from liquid biopsies replicate that from source tumor biopsies? In this review, we report the state of the art of extracellular RNA findings in uveal melanoma to shed light on the relationship between RNA dysregulations inside uveal melanoma cells and in the relative extracellular environments or patient bodily fluids.

## 2. Uveal Melanoma Biology

Uveal melanoma (UM) is the most common primary intraocular malignant tumor in adults and the most frequent non-cutaneous melanoma, though it is considered a rare disease [46]. Among all melanomas, UM represents about 3–5% of all cases, involving mainly the choroid (85–90%) and less frequently the ciliary body (5–8%) and the iris (3–5%) [47]. The disease shows a high mortality due to metastasis, which leads to death in up to 50% of UM patients within 10 years from UM diagnosis [48,49]; a median survival of 6 to 12 months after metastasis diagnosis has been reported [46,48]. Metastasis mostly develops in the liver (70–90%), though other organs may be involved, such as lungs (24–29%), bones (16–17%), skin (11–12%), and lymph nodes (11%) [50,51]. UM incidence is associated with ethnicity, age, and sex. It has been observed that the incidence of UM decreases following a north-to-south gradient in Europe, likely because of higher ocular pigmentation typical of populations living in southern countries, which protects the eyes from ultraviolet (UV) radiation [52]. Indeed, European incidence estimation in 2007 ranged from a minimum <2 cases per million in southern Italy and Spain to a maximum of >8 cases per million in Norway and Denmark, with an incidence of 6 cases per million in central Europe [52]. Similarly, USA incidence was estimated in the same period as 4.3 cases per million, a value that rose with increasing latitude [53,54]. The disease mostly affects older people, showing a median age at diagnosis of about 62 years; median age varies in different populations, with Asian patients showing lower values. UM is rare in children, and congenital disease is extremely rare [55].

UM etiology is still under investigation. Despite their common origin from melanocytes, UM and cutaneous melanoma (CM) are different diseases both for genetic alterations and biological behavior [56]. While it is clear that UV radiation is the major risk factor for CM [57], its contribution to UM pathogenesis is not well-established [58]. Some studies showed a weak positive association between UV exposure and UM development [59,60,61], while others did not confirm this evidence [62,63,64,65]. On the contrary, it has been reported that increased UV exposure may have a protective effect, since outdoor workers showed a decreased risk of UM development compared to indoor workers [58]. Nevertheless, lower melanin levels in eyes or skin have been associated with UM [61,62], suggesting that an involvement of UV radiation in UM may exist, even if it is considerably weaker than in CM.

Mutations of five genes have been described as the most common in UM: GNAQ (G protein subunit alpha q), GNA11 (G protein subunit alpha 11), BAP1 (BRCA1 associated protein 1), EIF1AX (eukaryotic translation initiation factor 1A X-linked), and SF3B1 (splicing factor 3b subunit 1) [66,67]. GNAQ and GNA11 mutations are considered an early event in UM, fostering cell proliferation by activating the mitogen-activated protein kinase (MAPK) pathway. BAP1 is a well-known tumor suppressor gene mapping on chromosome 3, frequently altered by monosomy in UM; BAP1 regulates genome stability, epigenetic modifications, transcription regulation, and response to DNA damage, and has been identified as the first predisposing gene among hereditary forms of UM. SF3B1 and EIF1AX mutations mainly occur in UM without chromosome 3 monosomy and are considered late events in UM carcinogenesis [68]. Another frequent genetic alteration in UM is the copy number variation of entire chromosome arms: the most common aberration is monosomy of chromosome 3, occurring in 21% to 56% patients and representing the most important prognostic factor, indicating a high risk of metastasis [48]. The gain of chromosome 8q is frequent in metastasis and is a biomarker of poor prognosis when it co-occurs with monosomy of chromosome 3 [67]. The loss of chromosome 1, including the entire chromosome or only a part of it, is associated with poor prognosis [48].

UM diagnosis is based on clinical examination by ophthalmologists. Several ancillary tests, including ultrasound biomicroscopy (UBM), optical coherence tomography (OCT), indocyanine green angiography (ICGA), color fundus photography, ultrasonography (USG), fundus fluorescein angiography (FFA), and fundus autofluorescence (FAF), can be performed to diagnose the tumor [69]. Patients may be asymptomatic or suffer from blurred vision, photopsia, floaters, and visual field loss [70]. Therapeutic approaches include enucleation or treatments aiming to preserve the eye, comprising radiation, surgical, and laser therapy [69,71,72].

RNA molecules are already considered useful tools for UM management. The transcriptome of UM tumors has been analyzed by gene expression profiling, leading to the identification of two profiles able to predict prognosis: Class 1 is associated with low-risk of metastasis, Class 2 with high risk of metastasis [73]. By analyzing the expression of 15 genes (12 discriminating and 3 control genes) in tumor biopsies, it is possible to easily and accurately classify patients as low or high risk, improving treatment efficacy [74]. The disadvantage of this application based on RNA molecules as prognostic biomarkers is the need of tumor biopsy, obtained either by fine needle aspiration or eye enucleation. On the contrary, extracellular RNAs could be a better option, since they can be collected from bodily fluids with low- or non-invasive procedures. The best option as a biomarker source is serum/plasma, which can be easily collected by blood sampling, with little or no pain for the patient. Since UM disseminates tumor cells exclusively by hematogenous spread [75], blood is a suitable source of biomarkers. Other very interesting fluids to analyze in UM patients are those contained in the eye chambers, namely vitreous humor and aqueous humor: these fluids are nearly in direct contact with the tumor, harvesting molecules secreted by it, and can be collected with invasive procedures which preserve the eye. Importantly, eye humors would be a preferable source of biomarkers compared to blood: since the eye is an isolated compartment of the body, UM-related biomarkers from ocular fluids would not be too diluted as they are in blood. On the other hand, blood has the great advantage of ease, low cost, and non-invasive sampling. Therefore, looking for UM biomarkers, it is necessary to investigate potential biomarkers from both blood and eye humors, aiming to identify the best compromise in terms of biomarker accuracy and non-invasive sampling.

Generally, the main challenge in ocular pharmacotherapies is related to drug delivery [76,77,78]. The traditional systemic chemotherapy option is not successful in UM because of ocular barriers that affect distribution of drugs in the eye after systemic administration. Additionally, checkpoint inhibitor immunotherapy fails to stop the progression of metastatic UM [79]. Therefore, radiotherapy with other adjuvant therapies, e.g., target miRNAs (with agomirs or antagomirs), would be of value for the management of UM, whenever the tumor is not extended and enucleation cannot be avoided [80]. Additionally, a modulation of miRNAs through administration of drugs can be hypothesized [41].

## 3. Cellular and Serum/Plasma miRNAs as Prognostic and Diagnostic Biomarkers of UM

Circulating miRNAs are ideal prognostic and diagnostic biomarkers because of several characteristics: (i) serum stability, (ii) non-invasiveness, and (iii) potential high specificity and selectivity. We explored on PubMed the latest findings on miRNA prognostic and diagnostic significance in UM from 2008 to 2021. We also looked at clinicaltrials.gov (accessed on 22 November 2021), but no trial regarding miRNAs and UM was found.

Several papers investigated the expression of miRNAs in UM tissues and demonstrated their role as regulators of molecular processes promoting cancer progression. Some of these miRNAs were also reported as dysregulated in serum, and consequently proposed as UM biomarkers. On the other hand, few studies specifically focusing on circulating miRNAs in UM are available (Table 1).

### 3.1. Serum/Plasma Circulating miRNAs

The first study in which a miRNA expression pattern was assessed in the blood of UM subjects was published in 2012 by Triozzi et al. This study is particularly interesting for pharmacological aspects, since the miRNA expression profile was monitored in the plasma of UM patients subjected to enucleation or brachytherapy during 33 weeks of adjuvant therapy with dacarbazine and interferon-alfa-2b, and after 6 months from their last treatment. MiR-199a was found to be highly expressed in the plasma of UM subjects, and its expression was down-regulated only after 17 weeks of monitoring, after 8 weeks of interferon-alfa-2b, while no changes were observed after two infusions of dacarbazine. At 17 weeks of monitoring, along with miR-199a, miR-126 was found to be down-regulated, while miR-16 and miR-106a expression levels increased. However, starting from week 25, these miRNAs returned to basal levels. The study by Triozzi et al. provided new findings about circulating miRNAs in UM patients; however, no data were provided by the authors about subject stratification based on risk of metastasis during follow-up [81].

A small study was published in 2014 by Achberger et al. and involved six UM patients. The authors reported that six “immuno-modulatory” miRNAs (miR-20a, miR-125b, miR-146a, miR-155, miR-181a, and miR-223) were up-regulated in the plasma of patients compared with healthy individuals; all miRNAs also showed increased levels at metastasis compared to the primary diagnosis, except for miR-181a down-regulation. In this study, the development of metastasis in UM cells was associated with decreased circulating CD3^−^CD56^dim^ Natural Killer cells, CD8^+^ and double-negative CD3^+^CD56^+^ Natural Killer T cells. In these subjects, ICOS^+^CD4^+^FoxP3^+^ T regulatory cells and CD11b^+^CD14^−^CD15^+^ myeloid suppressor cells increased at metastasis development compared to primary UM diagnosis. The authors concluded that dysregulation of miR-20a, miR-125b, miR-146a, miR-155, miR-181a, and miR-223 may have a prognostic value and help in developing new immunotherapeutic approaches [85].

In the same year, Eldh and coauthors analyzed miRNA expression in exosomes isolated from the liver circulation in patients with metastatic UM. Patients affected by metastatic UM showed a higher concentration of exosomes in the systemic circulation compared with healthy subjects. By analyzing exosomal miRNA expression, three clusters of miRNAs were identified in exosomes isolated from liver perfusate of five metastatic UM patients compared with exosomes of five cancer cell lines (lung, breast, CM, and mast cells): Cluster 1, including four miRNAs (miR-216a, miR-217, miR-129-5p, and miR-203), and Cluster 2, consisting of seven miRNAs (miR-9*, miR-125a-5p, miR-25, miR-125b, miR-335, miR-19a, and miR-9), showed almost the same expression between patients and exosomes of cancer cell lines used as control; Cluster 3 miRNAs (miR-370, miR-210, miR-320a, miR-124, miR-107, and miR-486-5p) were primarily expressed in exosomes of patients compared to exosomes of cancer cell lines used as control, where they were not detected, except for CM cells [82].

In 2015, our group analyzed miRNA expression profiles in vitreous, vitreous exosomes and serum from UM subjects and unaffected individuals. In this study, the TaqMan Low Density Array (TLDA) technology showed that only 66% of serum miRNAs were also present in vitreous, and serum, vitreous, and vitreal exosomes shared a common expression profile of 147 miRNAs. Validation through qPCR showed that miR-146a and miR-618 were significantly up-regulated in the serum of UM subjects compared with healthy controls; miR-146a and miR-618 were also dysregulated in vitreous and vitreous exosomes of UM subjects. MiR-146a, miR-21, and miR-34a were also up-regulated in tumor specimens of UM patients compared with unaffected eyes. Only miR-146a was significantly up-regulated in all analyzed matrices, and its increased levels were also confirmed in exosomes isolated from serum. Overall, this study suggested serum miRNAs as potential biomarkers for UM [12]. Russo et al. in 2016 confirmed the upregulation of miR-146a in tumor tissues and serum of UM patients compared with unaffected individuals [86].

In 2016, Triozzi et al. carried out tumor and plasma miRNA expression profiling in subjects with primary UM bearing monosomy of chromosome 3, a predictor of metastasis risk. The study showed significant plasma overexpression of miR-199a-5p, miR-223, and miR-92b in patients with a high risk of metastatic UM compared with normal subjects and UM patients with chromosome 3 disomy [89].

In 2019, Stark et al. identified a panel of six serum miRNAs particularly valuable for identification of benign uveal lesions (nevi) compared with localized or metastatic UM. The authors analyzed the expression of a panel of 17 CM-related miRNAs in UM cell lines, showing that all miRNAs are expressed in at least one of the six tested cell lines. Among these miRNAs, miR-211, highly expressed in CM, was confirmed as highly expressed in five out of six UM cell lines. In serum samples of patients with benign uveal lesions (nevi) or localized or metastatic UM, 11 miRNAs were detected. Particularly, six miRNAs (miR-16, miR-145, miR-146a, miR-204, miR-211, and miR-363-3p) were significantly up-regulated in localized UM compared with uveal nevi, while increased levels of all miRNAs except for miR-204 were observed in metastatic UM compared with uveal nevi. Only serum miR-211 was a good biomarker for discriminating metastatic UM from localized UM [83].

### 3.2. Cellular Expression of Serum/Plasma Dysregulated miRNAs

Literature data available to date suggest that alterations of miRNA expression in serum/plasma of UM patients may be used as prognostic or diagnostic biomarkers. The previously discussed studies reported dysregulation of 22 miRNAs (miR-106a, miR-107, miR-124, miR-125b, miR-126, miR-145, miR-146a, miR-155, miR-16, miR-181a, miR-199a, miR-204, miR-20a, miR-210, miR-211, miR-223, miR-320a, miR-363-3p, miR-370, miR-486-5p, miR-618, and miR-92b), among which miR-146a was identified as altered in four studies. In light of this evidence, we investigated the expression of these miRNAs within tumor cells.

The first study on 24 primary human UM samples was carried out in 2008 by Worley et al., where the authors performed a microarray profiling, validated through qPCR. Through unsupervised analysis, they identified two classes of tumor: Class 1 (low risk of metastasis) and Class 2 (high risk of metastasis) [93]. The significance analysis of microarrays (SAM) evidenced that Class 2 tumors were characterized by up-regulation of six miRNAs (let-7b, miR-199a, miR-199a*, miR-143, miR-193b, and miR-652). All these miRNAs were also significantly associated with the loss of chromosome 3, which, along with the classification as a Class 2 tumor, is a prognostic feature of metastatic UM [88,94]. A miRNA profiling in UM tumor samples bearing chromosome 3 alterations was carried out in 2016 by Venkatesan et al. The authors compared miRNA expression in patients with chromosome 3 monosomy and disomy. Profiling results were validated through qPCR, showing up-regulation of five miRNAs (miR-214, miR-146b, miR-143, miR-199a, and miR-134) in monosomic compared with disomic tumors. Moreover, miR-149* and miR-134 expression levels strongly correlated with liver metastasis risk [90]. The Cancer Genome Atlas (TCGA) UM dataset was analyzed by Vashishtha et al. in 2020. The authors identified a miRNA expression pattern in tumor samples of metastatic compared with non-metastatic patients, including the upregulation of miR-199a-5p, miR-708-5p, and miR-592 and the downregulation of miR-508-3p, miR-509-3p, miR-508-5p, miR-514a-3p, miR-506-3p, miR-509-3-5p, miR-513c-5p, miR-513a-5p, and miR-513b-5p [91]. In 2019, Falzone et al. reanalyzed miRNA expression profiles in the TCGA UM dataset. The authors concluded that miR-592 and miR-199a-5p, two members of the miR-506-514 cluster, were the most upregulated miRNAs in high-grade compared to low-grade UM tissues; furthermore, these miRNAs were associated with the overall survival of patients [37]. According to these reports, miR-199a upregulation in UM tissues is associated with a high risk of metastasis and chromosome 3 monosomy, suggesting an active role of this miRNA in UM progression. In the extracellular compartment, miR-199a upregulation was also observed in plasma of patients showing a high risk of metastasis, while its plasmatic down-regulation was induced by treatment.

In 2015, Venza et al. profiled miRNA expression in UM samples compared with healthy tissues, reporting decreased levels of miR-15a, miR-185, and miR-211, along with an increase of interleukin-10 receptor alpha (IL-10Rα), which was linked to the development and progression of both CM and UM [92]. No data about these miRNAs in serum of UM patients are available to date, except for miR-211 [83], which showed an increased expression in serum of patients affected by localized UM compared to uveal nevi and in metastatic compared to localized UM.

Smit et al., in 2019, analyzed UM patients classified into three groups based on metastasis risk (low-, intermediate-, and high-risk), evaluated according to several parameters, such as disease-free survival and mutation status. The authors found a specific expression pattern associated with high risk of metastasis: miR-132-5p, miR-151a-3p, miR-17-5p, miR-16-5p, and miR-21-5p were up-regulated, whereas miR-181b-5p, miR-101-3p, miR-378d, miR-181a-2-3p, miR-99a-5p, miR-let-7c-5p, miR-1537-3p, and miR-99a-3p were down-regulated in tissues of high-risk patients compared to intermediate- and low-risk patients [84]. Among these miRNAs, only increased levels of miR-16 were reported in plasma of treated UM patients [81]. MiR-21 was found to be over-expressed in exosomes isolated from the vitreous of UM subjects, as reported in our previous study [12].

A recent post-hoc bioinformatic analysis of the TCGA UM dataset identified a hub prognostic mRNA signature. The authors identified four miRNAs (miR-181b, miR-507, miR-548, and miR-181a) that were predicted to interact with the prognostic mRNAs. Survival analysis showed that downregulation of the four miRNAs predicted poor prognosis, being associated with poor survival [87]. Among these miRNAs, upregulation of miR-181a was observed in plasma of UM patients, compared with unaffected individuals, and in metastatic compared to non-metastatic UM patients [85].

In conclusion, we found a potential link between miRNAs altered in UM cells and miRNAs found dysregulated in the blood of UM patients. These findings suggest that these miRNAs not only can have a prognostic or diagnostic value, but may also beinteresting pharmacological targets for UM treatment.

### 3.3. Dysregulated Circulating miRNAs as Potential Pharmacological Targets

**Figure 1:**
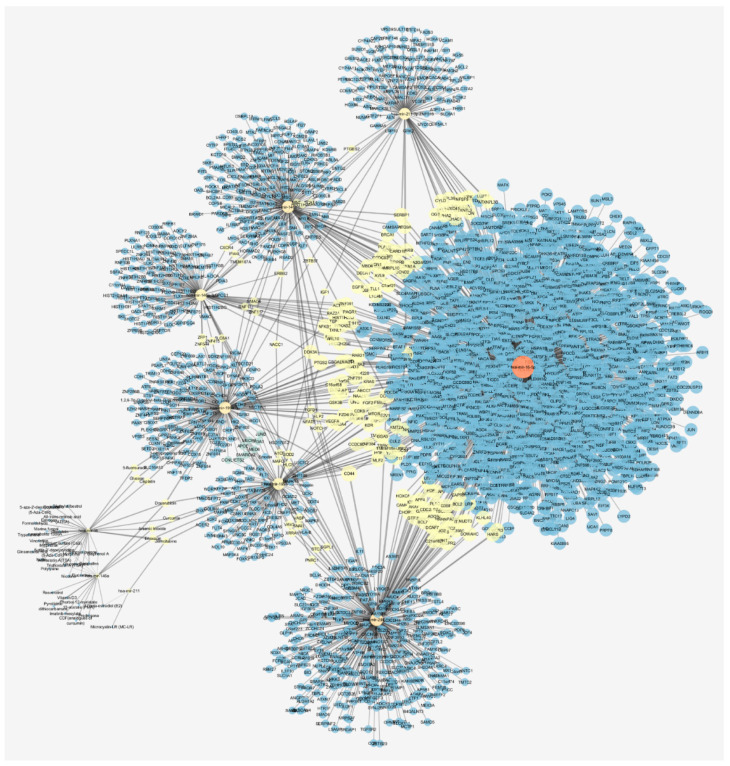
Gene–miRNA interactions were collected from three well-annotated databases: miRTarBase (https://mirtarbase.cuhk.edu.cn/~miRTarBase/miRTarBase_2022/php/index.php, 22 November 2021), TarBase v.8 (https://carolina.imis.athena-innovation.gr/diana_tools/web/index.php?r=tarbasev8%2Findex, 22 November 2021), and miRecords (http://c1.accurascience.com/miRecords/, 22 November 2021). The miRNA–compound interaction data were collected from SM2miR (http://www.jianglab.cn/SM2miR/, 22 November 2021) and PharmacomiR (http://www.pharmaco-mir.org/, 22 November 2021). Centrality network parameters (crucial for identification of nodes important for network stability) were calculated by using Cytoscape Network Analysis. Node dimension, which is proportional to closeness centrality values, and node color (blue < red) represents betweenness centrality values. Node thickness is proportional to edge betweenness values. Closeness centrality: distance of each node from all other nodes of the network; betweenness centrality: measure of the importance on a node basing on the shortest paths it is included into; edge betweenness: number of shortest paths between nodes that contain the edge.

Regarding the possibility to modulate miRNA levels through the administration of specific drugs, we predicted, through miRNET webserver (https://www.mirnet.ca/, accessed on 22 November 2021), a gene-miRNA-compound network (Figure 1), and several compounds were predicted to modulate miR-199a, miR-16, miR-211, and miR-146a (commonly dysregulated in UM) as shown in Table 2. Preclinical studies should be carried out to confirm the capability of these compounds to modulate miRNA expression in vitro and in vivo before further pharmaceutical or clinical development for these compounds as adjuvant therapies of UM.

## 4. Serum/Plasma Circulating Long RNAs in UM

### 4.1. Serum/Plasma Circulating Protein-Coding RNAs

The interest in circulating long RNAs arose back in the 1990s, when blood levels of TYR (tyrosinase) mRNA were investigated in CM and UM patients to detect circulating tumor cells (CTCs). Normal melanocytes should not circulate in blood; therefore, blood detection of a melanocyte-specific transcript such as TYR (coding an enzyme involved in melanin biosynthesis) should identify CTCs [95,96]. The first study exploring such an analysis was performed in 1991 by Smith et al. The authors analyzed TYR mRNA levels in blood circulating cells from patients affected by CM or other tumors and healthy individuals. Results were promising, showing TYR mRNA detection in four out of seven CM patients, while no patient affected by other tumors or healthy individuals gave positive results [95]. In 1993, Tobal et al. performed a similar experiment on UM patients. The authors collected circulating cells from blood samples of six UM patients and analyzed TYR mRNA levels in circulating cells, detecting it in two out of two metastatic patients and one out of four non-metastatic patients. This non-metastatic patient developed metastasis nine months after analysis, while no amplification was detected in control blood samples. This first study suggested the possible application of circulating RNAs as metastatic biomarkers; in particular, TYR mRNA was able to discriminate UM patients with metastasis from UM patients without metastasis [96]. Shortly after, the same group performed a similar analysis in a larger cohort including healthy individuals and patients affected by UM and CM, denying their previous findings: the presence of TYR mRNA (and therefore of melanocytes) in blood was shown to be very low in both early and advanced stages of UM [97]. Despite these conflicting results, many other studies focusing on TYR mRNA as a circulating biomarker in CM have been performed; however, there are still inconsistent results [98]. Concerning UM, a new study with a prospective purpose was published in 2005 [99]. Boldin et al. enrolled 41 non-metastatic UM patients and followed them for at least five years; at the time of UM diagnosis, 16 out of 41 patients (39%) showed a positive result for TYR mRNA. No association with tumor size and histology was observed, while a significant association was found between PCR data and five-year survival, with a higher risk of death for PCR positive patients. Association with metastasis was also investigated: not all positive patients (11 out of 16 PCR positive patients, 39%) developed metastases, and not all PCR negative patients survived [99]. Another study published in 2004 applied a more sensitive technique, real-time PCR, and included more mRNA biomarkers. Keilholz et al. analyzed circulating mRNA levels of three markers, namely TYR, MLANA (MelanA, previously known as MART-1), and PMEL (premelanosome protein, previously known as gp100), in the tissue and blood of CM patients, in the tissue from liver metastasis and the blood of UM patients, and in healthy individuals [100]. Concerning UM blood samples, TYR, MLANA, and PMEL were detected in 12.5%, 4%, and 4% primary UM samples, respectively, and in 60%, 77%, and 10% tumor samples, respectively. According to these results, PMEL cannot be applied as a disease biomarker given its high expression in healthy subjects, while TYR and MLANA showed a similar sensitivity. In two out of three patients who developed metastasis, CTC detection via real-time PCR preceded the diagnosis of metastasis [100]. The same research group performed another study, published in 2007, analyzing TYR and MLANA mRNA levels in 110 blood samples of UM patients over a six-year period. Real-time PCR results showed a positive signal for 11 patients (10%); in particular, TYR and MLANA were detected in five patients each, and both biomarkers were detected in one patient. The potential prognostic value of both biomarkers was investigated in univariate and multivariate analyses, showing that TYR and MLANA transcripts, both independently and combined, were associated with a high risk of metastasis developing within 2 years and predicted disease-free survival and overall survival [101]. Another study from the same group supported these findings in 2011, analyzing TYR and MLANA transcripts in blood samples of 68 metastatic UM patients. Specifically, 43 out of 68 patients (63%) showed a positive result for real-time PCR analysis: 31 were positive for TYR, 40 for MLANA, and 28 for both transcripts. In both groups with liver metastasis and liver plus extrahepatic metastasis, 67% of patients showed a positive PCR result, while only one patient with extrahepatic metastasis showed detectable levels of at least one biomarker. Patients showing negative PCR results had a longer progression-free survival and overall survival [102]. Finally, the same authors published another study in 2016 reporting data on 202 UM patients not undergoing any therapy [103]. TYR was detected in 1.1% of patients at diagnosis and in 2.2% at 30 min post-surgical manipulation; one patient showed a positive real-time PCR result preoperatively, and no one showed one postoperatively. MLANA gave a positive result in 10.9% of the patients at diagnosis and in 13.9% at 30 min post-surgery; 10% of preoperative and 17.5% postoperative patients were positive for MLANA. No statistical difference in TYR and MLANA expression was found between preoperative and postoperative patients, in agreement with previously discussed studies [103]. Other research groups also investigated CTC detection in blood of UM patients by using PCR. In 2007, Callejo et al. reported undetectable levels of TYR and MLANA transcripts in the blood of healthy individuals and non-melanoma patients, including patients with small choroidal nevi, uveitis, patients enucleated for blind painful eye, trauma, or non-melanocytic tumors [104]. Moreover, the authors were able to detect the analyzed biomarkers both at the time of diagnosis and several months to years later; this evidence was also confirmed in a small subgroup of irradiated (3) or enucleated (6) patients. Enucleated patients showed no clinical sign of recurrence or metastasis; therefore, the authors hypothesized the presence of micrometastases, which could represent the source of CTCs. Overall, CTCs were found in 87.5% of examinations performed throughout the study; MLANA showed higher sensitivity than TYR, being detectable in 47.1% of all examinations, while TYR was detected only in 7.6% of examinations; detection of both biomarkers occurred in 45.3% of examinations [104]. In 2010, Pinzani et al. analyzed TYR mRNA levels using real-time PCR in the whole blood of 41 UM patients followed for 5 years [105]. TYR was not detected in all negative control specimens, except for two pre-operative and one post-operative sample. Among UM patients, 20 out of 41 (49%) showed detectable levels of TYR mRNA in at least one of three pre-therapeutic samples. TYR levels positively correlated with tumor size, with patients affected by a small tumor (7) showing undetectable levels of TYR transcript for the entire period of follow-up (17–32 months). This study also reported a positive association between TYR levels and disease-free survival and overall survival [105].

Taken together, the discussed studies showed that detection of CTCs in patient blood by PCR-based analysis of RNA biomarkers may be applied in clinics for prognostic purposes. RNA-based biomarkers (above all TYR and MLANA, the most investigated transcripts) may be used to predict poor prognosis (in terms of disease-free and overall survival) or to assess the risk of metastasis development. These data also confirm that metastatic UM has to be considered a systemic disease, despite the fact that the majority of metastases develop in liver; the presence of CTCs in blood also suggests that metastases develop through hematogenous dissemination. Finally, comparison between pre-operative and post-operative levels of RNA-based biomarkers (and, indirectly, of CTCs) showed no significant difference, suggesting that tumor resection should not be considered a source of CTC dissemination.

### 4.2. Serum/Plasma Circulating lncRNAs

In the last few decades, researchers have focused their attention on the role of lncRNAs in carcinogenesis [106]. Moreover, lncRNAs were found to be involved in the regulation of a plethora of cancer-related processes in UM [107,108,109,110,111,112,113,114]. Given the important contribution of lncRNAs to cancer progression, their potential application as circulating biomarkers has been investigated. The goal of diagnosing tumors by using non-invasive diagnostic biomarkers is very attractive. To date, many papers have been published reporting the differential expression of lncRNAs in biological fluids of patients affected by several cancers compared with healthy individuals [115]. Regarding UM, no report is available, likely because of both the rarity of this cancer and the recent interest in lncRNAs. To our knowledge, the only mention of extracellular lncRNAs in UM patients is our recent study on the role of LINC00518 (long intergenic non-protein coding RNA 518) in UM carcinogenesis. We observed a strong upregulation of LINC00518 in tumor tissues compared with adjacent normal tissues from the same patients; successively, we analyzed vitreous humor, serum, and exosomes isolated from both vitreous and serum collected from UM patients and healthy individuals; however, we did not detect this lncRNA in any sample [109]. This evidence suggests that LINC00518 plays a crucial role in tumor progression and is retained within tumor cells; however, it cannot be used as a circulating biomarker. Biomarkers are not always causally involved in pathogenetic processes; they may also be an effect of the disease (just as hyperglycemia is both an effect and a diagnostic biomarker for diabetes).

## 5. Circulating RNAs in Ocular Fluids

Aiming to identify RNA biomarkers for UM, the possibility to analyze the fluids in direct contact with the tumor is very attractive. Ocular fluids, namely aqueous and vitreous humor, are an optimal source of UM biomarkers because of the special conformation of the eye, which is an isolated district of the human body. Ocular fluids would thus allow the collection of biomarkers specific of the eye, which would be more concentrated than in blood. Blood represents the most commonly used source of biomarkers for several reasons, such as: (i) it can be collected with easy, low cost, and non-invasive sampling procedures; (ii) it reaches every compartment, collecting biomarkers from the entire body. The latter propriety represents both an advantage and a disadvantage, because biomarkers specific for a certain disease or condition are diluted in a large volume and are mixed with many other biomarkers from several source tissues. In this context, ocular fluids would overcome this issue, but they also show a great disadvantage, that is the invasive and unpleasant sampling procedure. Some reports in literature analyzed ocular fluids searching for biomarkers of a different chemical nature for various ocular diseases. Among protein biomarkers, VEGFA protein expression was investigated in vitreous and aqueous humor of UM patients [116,117]; similarly, S-100 was detected in ocular fluids [118] and HLA in aqueous humor [119] of UM patients. VEGFA is implicated in the pathophysiology of UM, and high serum concentrations of VEGFA were observed in metastatic UM patients [120]. VEGFA showed high levels in the vitreous and anterior chamber fluid of UM patient eyes, with the latter event being positively significantly correlated with tumor diameter and tumor height [116,117]. The major sources of VEGFA in the ocular fluids of UM patients are retina and tumor cells [117]. Even though the use of anti-VEGF agents to handle UM is controversial, it has been demonstrated that bevacizumab significantly prevented growth and metastasis development after UM cells’ inoculation into the choroid in mouse models of UM [121]. Recently, Tura et al. demonstrated that ranibizumab has a more potent and persistent suppressive activity on UM cells compared to bevacizumab [122]. It is worth noting that anti-VEGF agents used in clinical practice, such as ranibizumab, bevacizumab, and aflibercept, are considerably different in terms of molecular interactions when they bind VEGF [123]. Among the nucleic acid biomarkers, cell free DNA [124], RNA, and miRNAs showed measurable concentrations in aqueous humor of retinoblastoma patients [125]. Moreover, miRNA biomarkers were found in vitreous humor of patients affected by several ocular diseases, such as retinal detachment with different grades of proliferative vitreoretinopathy [40], idiopathic epiretinal membrane, and macular hole [126]. Odriozola et al. suggested a possible application of miRNAs detected in the vitreous humor of corpses as potential tools useful to determine time of death [127]. Similar studies have also been performed in UM (Table 1): the first detection of circulating miRNAs in vitreous humor was reported in 2013 [38], when our group analyzed miRNA expression in the vitreous humor of patients affected by UM, retinal detachment, and macular hole. Overall, 94 miRNAs were detected in vitreous and subdivided into three groups: highly expressed (HE), normally expressed (NE), and lowly expressed (LE) miRNAs. Vitreous levels of these miRNAs were compared with serum levels, showing that some miRNAs are mainly found in vitreous. Some HE miRNAs (miR-628, miR-302c, miR-639, miR-211, and miR-9) were significantly enriched in vitreous compared to serum, with more than 100-fold higher expression values; similarly, some NE miRNAs (miR-452, miR-9*, miR-214, miR-184, and miR-125a-3p) were greatly upregulated in vitreous compared to serum. On the contrary, some HE and NE miRNAs showed strongly reduced levels in vitreous compared to serum (miR-223, miR-24, miR-484, miR-191, miR-92a, miR-30c, miR-30-5p, miR-20a, miR-150, miR-16, miR-451, and miR-93*). UM samples showed a higher general expression of miRNAs compared with the other vitreous samples. Specifically, miR-26a, miR-34a, miR-146a, and miR-532-5p showed the highest expression in UM vitreous compared to the vitreous samples of the other analyzed diseases, suggesting their specific involvement in UM [38]. In 2015, our group performed another study on UM patients, analyzing miRNA expression in vitreous humor, serum, and exosomes isolated from vitreous [12]. Results showed miRNAs detected in vitreous and in exosomes isolated from vitreous, respectively. About 90% of exosomal miRNAs were also detected in vitreous humor, suggesting that the majority of vitreal miRNAs are included in nanovesicles. Commonly dysregulated miRNAs showed a significant positive expression correlation, suggesting that vitreal miRNA alteration in UM eyes is sustained by modifications of exosomal cargo and, therefore, is actively influenced by tumor cell activity. Among differentially expressed miRNAs, miR-146a, miR-21, and miR-34a showed increased expression in both vitreous and vitreal exosomes of UM patients compared with unaffected individuals, while miR-618 was downregulated in vitreous and upregulated in vitreal exosomes. Upregulation of miR-146a was also observed in serum and exosomes isolated from serum, and upregulation of miR-618 was confirmed in serum. MiR-146a, miR-21, and miR-34a also showed increased expression in formalin-fixed, paraffin-embedded (FFPE) UM samples compared with choroidal melanocytes from unaffected eyes [12]. Smith et al. reported the upregulation of miR-21 in UM tissues from patients at high risk of metastasis compared to intermediate- and low-risk UM subjects [84]. These results showed that, regarding miR-146a, miR-21, and miR-34a, alteration of expression in UM tissues matches variation in the extracellular compartment; however, this does not always happen, since alteration of miR-618 in vitreous and vitreous exosomes was not confirmed in UM tissues [12]. Similarly, our group reported the strong upregulation of the lncRNA LINC00518 in UM tissues compared with normal adjacent tissues, demonstrating its role in migration and metastatic-related processes in UM cell lines. Expression of LINC00518 was also investigated in vitreous, serum, and exosomes isolated from both vitreous and serum, but no detectable signal was observed [109].

## 6. Biological Meaning of Extracellular RNAs in UM

The main question addressed by this review was “How much do extracellular RNAs from UM patients represent a molecular snapshot of pathological status of UM donor cells?” Based on literature data, the overlap between cellular and extracellular dysregulated RNAs in UM is partial.

This obvious conclusion is the result of the different molecular strategies that normal and cancer cells exploit to control RNA secretion. RNA molecules circulating in vitreous or blood from UM patients are a mixture of (a) highly expressed cellular miRNAs, which are passively trapped in apoptotic bodies and microvesicles, or pass through the endosomes for an osmotic-like effect and flow within the exosomes; (b) selectively secreted RNAs whose function inside the cell could be unnecessary in cancerous conditions, but that are suitable to modify the behavior of surrounding cells, thus favoring cancer progression.

Although the encapsulation of miRNAs inside the exosomes would be a selective process regulated by specific protein mechanisms and sequence motifs on miRNAs [128,129,130], some findings suggest that a portion of miRNAs is passively delivered through exosomes depending on their intracellular concentration. On the other hand, miRNAs with a critical role for cell functioning tend to be selectively retained by cells and nearly absent in the extracellular environment regardless of their concentration [131,132,133]. Moreover, the presence of RNA fragments inside exosomes suggests a relationship between RNA turnover and exosome packaging [4]. Exosomes are involved in the homeostasis of intracellular RNA by inducing the release of misfolded or degraded RNAs [134]. RNA degradation and export to extracellular vesicles are strongly related; a crosstalk between endosomes originating exosomes and the lysosomal degrading pathway does exist [135].

The combination of these conflicting mechanisms promoting RNA release would lead to a heterogeneous population of extracellular RNAs not necessarily related to those from the cancer cells secreting them.

However, other objective circumstances would add further complexity layers. Transformed melanocytes from UM could secrete extracellular RNAs that would flow inside the vitreal chamber through retinal detachment, commonly occurring in UM patients [12]. In the vitreous humor of the eye chamber, RNA secreted by other cell types different from melanocytes (e.g., retina cells, phagocytes, hyalocytes of Balazs) could be present. The presence of a growing mass of cancerous melanocytes under the retina multilayer would directly or indirectly influence all the cells around it, which, in turn, could modify the RNA delivery into the vitreal chamber. In this way, RNAs circulating in vitreous would be a concoction of different healthy, cancer-conditioned, and transformed donor cells: accordingly, UM-related RNA signals would tend to be partially concealed. Furthermore, a part of RNA carriers in vitreous could be conveyed, by passing through the blood–ocular barrier, to the systemic circulation, where they mingle with extracellular RNAs secreted by various cell types, including endothelial and immune cells. UM, as well as many other tumors, can evade immune surveillance through signals that favorably mold the tumor microenvironment and promote immune-suppression [136]. Micro- and nano-vesicles coming from activated immune cells could play an important role in changing the concentration of circulating RNAs. This observation strongly suggests that a considerable part of RNAs altered in the blood of UM patients could derive from different activated regulatory immune cells. In agreement with such a phenomenon, it should not be surprising that a portion of UM biomarker RNAs, uncorrelated to UM biology, also has a critical functional role in the differentiation and activation of immune cells [137,138,139].

It is conceivable that the RNA populations present in UM bodily fluids, primarily in blood, would represent the complex molecular mixture of different cellular sources (e.g., transformed melanocytes, healthy cells, and immune cells). For instance, tyrosinase mRNA, highly expressed in cancerous melanocytes, may circulate within apoptotic bodies, as already demonstrated for CM [140]. Levels of miR-618, unchanged in UM tissues, increased in vitreal exosomes and serum from the same patients: miR-618 upregulation could be induced by active or passive secretion mechanisms by resident plasmacytoid dendritic cells in the eye, where miR-618 induces IFNα secretion during anticancer response [141,142,143,144]. Several papers reported the upregulation of miR-146a in UM (but also in CM), both in tissues and blood [12,38,83,86,145]; moreover, we previously found increased levels of miR-146a in exosomes from vitreous and serum of UM patients [12,38]. MiR-146a plays a critical role in melanocyte transformation because it is able to enhance tumor growth and could be involved in modulation of sensitivity to Natural Killer cell lysis [146,147]. Accordingly, UM cells could passively or actively, through exosomes, secrete miR-146a in excess into vitreous humor and blood. MiR-146a is also a negative regulator of T-cell activation in the melanoma microenvironment, suggesting that also immune cells could contribute to the miR-146a exosomal increase observed in UM blood [148].

In conclusion, extracellular RNAs circulating in bodily fluids of UM patients do not represent a mirror of RNA dysregulation occurring in cancerous tissues, with rare exceptions (e.g., miR-146a and tyrosinase), but rather they are the complex outcome of a systemic dysfunction that may induce active and passive mechanisms of RNA delivery from a plethora of cell types.

## Figures and Tables

**Table 1:** miRNAs showing altered expression in extracellular compartments and tissues in UM patients.

miRNAs	Plasma	Serum	Exosomes from Liver Perfusate	Vitreous Humor	Tissue
miR-106a	up after interferon-alfa-2b therapy [81]				
miR-107			up in patients [82]		
miR-16	up after interferon-alfa-2b therapy [81]	up in localized and metastatic UM [83]			up in high-risk patients [84]
miR-124			up in patients [82]		
miR-125b	up in UM and metastatic patients [85]				
miR-126	down after interferon-alfa-2b therapy [81]				
miR-145		up in localized and metastatic UM [83]			
miR-146a	up in UM and metastatic patients [85]	up in serum and serum exosomes of UM patients [12]		up in vitreous humor and vitreal exosomes of UM patients [12]	up in UM tissue [12]
		up in localized and metastatic UM [83]		highest expression in UM patients [38]	
		up in serum of UM patients [86]			up in UM tissue [86]
miR-155	up in UM and metastatic patients [85]				
miR-16	up after interferon-alfa-2b therapy [81]	up in localized and metastatic UM [83]			up in high-risk patients [84]
miR-181a	up in UM patients; down in metastatic patients [85]				down in patients with poor survival [87]
miR-199a	down after interferon-alfa-2b therapy [81]				up in patients with high risk of metastasis [88]
	up in patients with high risk of metastasis [89]				up in monosomy of chromosome 3 [90]
					down in metastatic patients [91]
					up in high grade UM [37]
miR-20a	up in UM and metastatic patients [85]				
miR-204		up in localized UM [83]			
miR-21				up in vitreous humor and vitreal exosomes of UM patients [12]	up in UM tissue [12]
					up in high risk of metastasis [84]
miR-210			up in patients [82]		
miR-211		up in localized and metastatic UM [83]			down in UM tissue [92]
miR-223	up in UM and metastatic patients [85]				
	up in patients with high risk of metastasis [89]				
miR-26a				highest expression in UM patients [38]	
miR-320a			up in patients [82]		
miR-34a				up in vitreous humor and vitreal exosomes of UM patients [12]	up in UM tissue [12]
				highest expression in UM patients [38]	
miR-363-3p		up in localized and metastatic UM [83]			
miR-370			up in patients [82]		
miR-486-5p			up in patients [82]		
miR-532-5p				highest expression in UM patients [38]	
miR-618		up in serum of UM patients compared to unaffected individuals [12]		down in vitreous and up in vitreal exosomes of UM patients [12]	
miR-92b	up in patients with high risk of metastasis [89]				

**Table 2:** Compounds able to modulate the expression of miR-199a, miR-16, miR-211, and miR-146a. Degree and eccentricity values (centrality metric parameters necessary for the identification of nodes important for network stability) were calculated by using Cytoscape Network Analysis. Degree: number of connections of each node with the others; eccentricity: the maximum distance from a node to any other node of the network.

Degree	Eccentricity	Name
4	4	Doxorubicin
1	6	Pyrrolidine dithiocarbamate
1	6	Resveratrol
2	4	5-fluorouracil
1	6	5-aza-2′-deoxycytidine (5-Aza-CdR)
1	6	5-aza-2′-deoxycytidine (5-Aza-CdR) + trichostatin A(TSA)
2	4	Cisplatin
1	6	Bortezomib
1	6	Calcium sulfate (CaS)
3	4	Enoxacin
2	4	Curcumin
3	4	Arsenic trioxide
1	6	Estrogen
1	6	All-trans-retinoic acid (ATRA)
1	6	Melphalan
2	6	Nicotine
2	4	Glucose
1	6	Trypaflavine
1	6	Marine fungal metabolite 1386A
2	6	Morphine
2	6	Trichostatin A (TSA)
2	6	Benzo(a)pyrene
2	6	Glucocorticoid
1	6	Formaldehyde
1	6	Ginsenoside Rh2
1	6	Polylysine
1	6	Diethylstilbestrol
3	4	Gemcitabine
2	6	Bisphenol A
1	6	Fluoxetine
1	6	Genistein
1	6	Vincristine
1	6	Tamoxifen
1	6	Nutlin
2	6	17beta-estradiol (E2)
1	6	Microcystin-LR (MC-LR)
1	6	CDF(analogues of curcumin)
1	6	Imatinib mesylate
1	6	Vitamin D3
1	6	Phorbol 12-myristate 13-acetate (PMA)
1	6	Prednisone
1	6	1,2,6-Tri-O-galloyl-beta-D-glucopyranose
1	6	3-nitropropionic acid (3-NPA)
1	6	Activin A
1	6	Oltipraz
1	6	Paclitaxel
1	4	Ethanol
1	4	Emodin
1	4	Heparin
